# Pesticidal Plant Extracts Improve Yield and Reduce Insect Pests on Legume Crops Without Harming Beneficial Arthropods

**DOI:** 10.3389/fpls.2018.01425

**Published:** 2018-09-28

**Authors:** Yolice Tembo, Angela G. Mkindi, Prisila A. Mkenda, Nelson Mpumi, Regina Mwanauta, Philip C. Stevenson, Patrick A. Ndakidemi, Steven R. Belmain

**Affiliations:** ^1^Lilongwe University of Agriculture and Natural Resources, Lilongwe, Malawi; ^2^Nelson Mandela African Institution of Science and Technology, Arusha, Tanzania; ^3^Jodrell Laboratory, Royal Botanic Gardens, Richmond, United Kingdom; ^4^Natural Resources Institute, University of Greenwich, Chatham Maritime, Kent, United Kingdom

**Keywords:** pest control, pesticidal plants, botanical products, ecosystem services, agro-ecological intensification, sustainable agriculture

## Abstract

In the fight against arthropod crop pests using plant secondary metabolites, most research has focussed on the identification of bioactive molecules. Several hundred candidate plant species and compounds are now known to have pesticidal properties against a range of arthropod pest species. Despite this growing body of research, few natural products are commercialized for pest management whilst on-farm use of existing botanically-based pesticides remains a small, but growing, component of crop protection practice. Uptake of natural pesticides is at least partly constrained by limited data on the trade-offs of their use on farm. The research presented here assessed the potential trade-offs of using pesticidal plant extracts on legume crop yields and the regulating ecosystem services of natural pests enemies. The application of six established pesticidal plants (*Bidens pilosa, Lantana camara, Lippia javanica, Tephrosia vogelii, Tithonia diversifolia*, and *Vernonia amygdalina*) were compared to positive and negative controls for their impact on yields of bean (*Phaseolus vulgaris*), cowpea (*Vigna unguiculata*), and pigeon pea (*Cajanus cajan*) crops and the abundance of key indicator pest and predatory arthropod species. Analysis of field trials showed that pesticidal plant treatments often resulted in crop yields that were comparable to the use of a synthetic pesticide (lambda-cyhalothrin). The best-performing plant species were *T. vogelii, T. diversifolia*, and *L. javanica*. The abundance of pests was very low when using the synthetic pesticide, whilst the plant extracts generally had a higher number of pests than the synthetic but lower numbers than observed on the negative controls. Beneficial arthropod numbers were low with synthetic treated crops, whereas the pesticidal plant treatments appeared to have little effect on beneficials when compared to the negative controls. The outcomes of this research suggest that using extracts of pesticidal plants to control pests can be as effective as synthetic insecticides in terms of crop yields while tritrophic effects were reduced, conserving the non-target arthropods that provide important ecosystem services such as pollination and pest regulation. Thus managing crop pests using plant secondary metabolites can be more easily integrated in to agro-ecologically sustainable crop production systems.

## Introduction

The search for novel pest control products from plants continues to grow, but not always with clear outcomes and benefits (Isman and Grieneisen, 2013). However, there are many candidate plant species with known pesticidal properties where much is already known about their chemistry and efficacy under laboratory conditions that could be rapidly developed in to new products (Stevenson et al., 2017). Isman (2017) has argued that increasing farmer use of natural pesticides needs research directed at the practical application of such products under complex agro-ecological conditions, particularly understanding how different pesticidal plant species perform when applied to different crops under different growing conditions. Furthermore, their effects against target and non-target species, safe use and overall socio-economic and agro-ecological benefits need work. Only through their evaluation under field conditions can the evidence for more widespread adoption of natural pest control products be found, particularly as natural compounds are often not as effective as current synthetic pesticides (Casida, 1980). Using unrefined plant extracts for pest control has several advantages in terms of preventing the development of insecticide resistance due to the usual presence of several bio-active compounds, their low persistence in the environment and their generally low cost of use, particularly for smallholder farmers with limited income (Angioni et al., 2005; Caboni et al., 2006; Isman, 2008). However, disadvantages include variable efficacy, and low toxicity and persistence against target pests, which is partly due to the rapid breakdown of bio-active compounds, for example through photodegradation, and due to such extracts easily washing off when it rains. Consumers and policy makers are demanding reduced synthetic inputs in food production, and practices that support agro-ecological intensification and pesticidal plant products may be well suited to this vision (Grzywacz et al., 2014; Sola et al., 2014; Pavela, 2016).

An important trade-off to consider in the fight against arthropod crop pests using plant secondary metabolites, is the impact of crop protection strategies on ecosystem services. Pollination and natural pest regulation by arthropods are affected negatively by the use of synthetic pesticides (Rundlöf et al., 2015; Potts et al., 2016). The value of natural suppression of aphids on soya bean was valued at US$239 million in four US states (Landis et al., 2008), so the benefits of natural pest regulation can be measured in terms of environmental and economic value. Natural pest control is an ecosystem service that can be augmented and sustained by natural or manipulated agro-ecosystems (Gurr et al., 2016, 2017) as well as local land management practices which can impact on availability of pollinators on legume crops such as pigeon pea (Lautenbach et al., 2012). Many factors appear to be causing pollinator decline; however, the increasing use of synthetic pesticides is one of the primary causes (Potts et al., 2016), and policies that facilitate more environmentally benign approaches are required for sustainable agriculture (Dicks et al., 2016). Although some research has been conducted on the impact of pesticidal plant use on non-target arthropods (Mkenda et al., 2015; Mkindi et al., 2017), this remains a neglected area of research that needs further investigation to understand the trade-offs of using more plant-based pest control products.

The research presented here established whether crude extracts of six cosmopolitan pesticidal plant species have potential as the basis of biopesticides on different legume crops and to understand the impacts of using pesticidal plants on non-target arthropods.

## Materials and methods

### Study site

The study was conducted at field sites in Tanzania and Malawi over three years where common bean (*Phaseolus vulgaris*) was grown during 2015, cowpea (*Vigna unguiculata*) was grown during 2016 and pigeon pea (*Cajanus cajan*) was grown during 2017 cropping seasons. Field trials were carried out at Nelson Mandela African Institution of Science and Technology, Arusha, Tanzania (Latitude 3°24′S Longitude 36°47′E and at Lilongwe University of Agriculture and Natural Resources, Bunda, Malawi (Latitude 14°11′S Longitude 33°46′E). In Tanzania, the location was at an elevation of 1,168 masl with a mean annual rainfall of 1,200 mm, mean maximum temperature of 21.7°C and mean minimum temperature of 13.6°C. For Malawi, the location was at an elevation of 1,100 masl with a mean annual rainfall of 700 mm, mean maximum temperature of 29°C and mean minimum temperature of 17°C.

### Experimental design

The farm land where field trials took place was disc harrowed and ridged prior to planting. The common bean seeds used for planting were of the variety Lyamungo 90 in Tanzania and Kalima in Malawi. In Tanzania, the seeds were planted at a spacing of 50 cm between rows and 20 cm within rows in 5 × 5 m plots which were 1 m apart. In Malawi, beans were planted 75 cm part with 2 rows of beans on each ridge, with rows spaced 10 cm apart and ridges 30 cm apart and plants spaced 10 cm within rows in 5 × 5 m plots which were 1 m apart. The cowpea seeds used for planting were of the variety Raha1 in Tanzania and Mkanakaufiti in Malawi. In Tanzania, the seeds were planted at a spacing of 50 cm between rows and 20 cm within rows in 5 × 5 m plots which were 1 m apart. In Malawi, cowpeas were planted on ridges of 75 cm apart with 1 row on each ridge, spaced at 20 cm within rows in 5 × 5 m plots which were 1 m apart. The pigeon pea seeds used for planting were of the variety Mali in Tanzania and Mthawajuni in Malawi. In Tanzania, the pigeon pea seeds were planted at a spacing of 75 cm between rows and 30 cm within rows in 5 × 5 m plots which were 2 m apart. In Malawi, pigeon pea were planted at a spacing of 75 cm between rows and 60 cm within rows in 5 × 5 m plots which were 2 m apart. Diammonium phosphate fertilizer was applied according to manufacturer's instructions during planting of the seeds. Trials were inspected each week with *ad hoc* hand weeding carried out as necessary. The experimental layout was a randomized complete block design, and the treatments were replicated on four blocks (Anderson and McLean, 1974).

### Plant species collection and processing

Fresh leaves of *Tephrosia vogelii* (Hook f.) (Fabales: Fabaceae), *Vernonia amygdalina* (Delile) (Asterales: Asteraceae), *Lippia javanica* (Burm.f.) Spreng. (Lamiales: Verbenaceae), *Tithonia diversifolia* (Hemsl.) A. Gray (Asterales: Asteraceae), *Bidens pilosa* L. (Asterales: Asteraceae), and *Lantana camara* L. (Lamiales: Verbenaceae) were collected from different locations around Hai District, Tanzania and Mitundu District, Malawi (voucher specimens and GPS coordinates lodged at Nelson Mandela African Institution of Science and Technology, Arusha, Tanzania and Lilongwe University of Agriculture and Natural Resources, Bunda, Malawi). Common bean field trials were carried out with four plant species (*L. javanica, T. diversifolia, T. vogelii*, and *V. amygdalina* in Tanzania and *L. camara, T. diversifolia, T. vogelii*, and *V. amygdalina* in Malawi) whilst cowpea and pigeon pea trials were carried out with all six plant species. These plant species were chosen due to their wide abundance around farms, roadsides, and bushland, their familiarity to farmers and considerable existing knowledge on their efficacy, bioactive constituents and safety (Ganjian et al., 1983; Pereira et al., 1997; Gu et al., 2002; Adedire and Akinneye, 2004; Kawuki et al., 2005; Viljoen et al., 2005; Ambrósio et al., 2008; Asawalam et al., 2008; Mujovo et al., 2008; Oyewole et al., 2008; Bagnarello et al., 2009; Gadzirayi et al., 2009; Adeniyi et al., 2010; Madzimure et al., 2011; Belmain et al., 2012; Stevenson et al., 2012). To ensure uniformity, the leaves from each seasonal collection were mixed together for each species before drying. Leaves were dried under shade for a week and then crushed using a mill and sieved into a fine powder. Powders were stored in black plastic bags in dark, dry conditions until required. New plant material was harvested each year.

### Field treatments

Data on the effect of plant extract concentration has been reported with expected dose response trends on crop yield when applying plant treatments at 0.1, 1.0, and 10% w/v (Mkindi et al., 2017). All data presented here are based on the application of 10% w/v as this was determined to be the most effective concentration for reducing insect damage and maintaining high crop yield (Mkindi et al., 2017). In making 10% extracts, 1 kg of plant powder was weighed and added to 10 L water to extract at ambient temperature (20 ± 5°C) for 24 h. In all cases 0.1% soap was added to the water during extraction as detergent increases the extraction efficiency of non-polar compounds from plant material (Belmain et al., 2012). Extracts were kept in 10 L buckets with lids in the shade and, shortly before application, filtered twice through a coarse and then fine cloth to remove all plant material that may inadvertently clog the sprayer. Negative controls consisted of water +0.1% soap and water only. The positive control in all trials was synthetic pesticide Karate 5 EC (lambda-cyhalothrin pyrethroid, Syngenta) which was applied as per the manufacturers' instructions (20 g/ha). All treatments were replicated across four blocks and were sprayed throughout the growing season at an interval of 7 days starting 1 week after crop plant emergence. A 15 L knapsack sprayer was used to apply the various treatments, and the sprayer was thoroughly cleaned with soap and water prior to being re-filled with another formulation for application.

### Sampling for presence of arthropod pest and beneficial species

All assessments were carried out the day before treatments were to be sprayed. Three inner rows from each plot were selected for sampling. Five plants in the selected three middle rows were visually examined to record the number of each arthropod type. Preliminary work indicated that a number of pest and beneficial species were documented on legume crops; however, many were only present infrequently or in low numbers. Furthermore, to assist with data collection, enumerators focussed on more obvious life stages and relatively larger insects. For example, parasitoids and larval forms of hoverflies and lacewings were not monitored due to their small size and difficulty to assess quickly. Thus indicator pest and beneficial species were chosen for monitoring that were easily identified and observed to be abundant throughout the cropping season. Thus the main target pest species evaluated were aphids (*Aphis fabae* Scopoli) (Hemiptera: Aphididae), bean foliage beetle (*Ootheca mutabilis* (Schönherr) and *O. bennigseni* Weise) (Chrysomelidae: Galerucinae) and flower beetle (*Epicauta albovittata* Gestro and *E. limbatipennis* Pic) (Coleoptera: Meloidae). The target predatory species were lady beetles (adults and larvae) (Coccinellidae), spiders (Araneae), and hoverflies (adults only) (Syrphidae). Due to often very high numbers, a categorical index was used to assess aphid abundance, where 0 = None; 1 = A few scattered individuals; 2 = A few isolated colonies; 3 = Several isolated colonies; 4 = Large isolated colonies; and 5 = Large continuous colonies (Mkindi et al., 2017). For analytical purposes, this index was used as a proxy to report aphid numbers. For all other arthropod species, the actual number of individuals was counted.

### Data analysis

Differences among treatments in insect abundance and bean yield were assessed by analysis of variance (ANOVA) and Tukey's *post-hoc* Honestly Significant Difference (HSD) test to separate the means at the 95% confidence interval. Analyses were performed in XLSTAT version 2015.1.01 (Addinsoft, Paris, France). Datasets are available on request.

## Results

### Arthropod abundance

**Figure 1:**
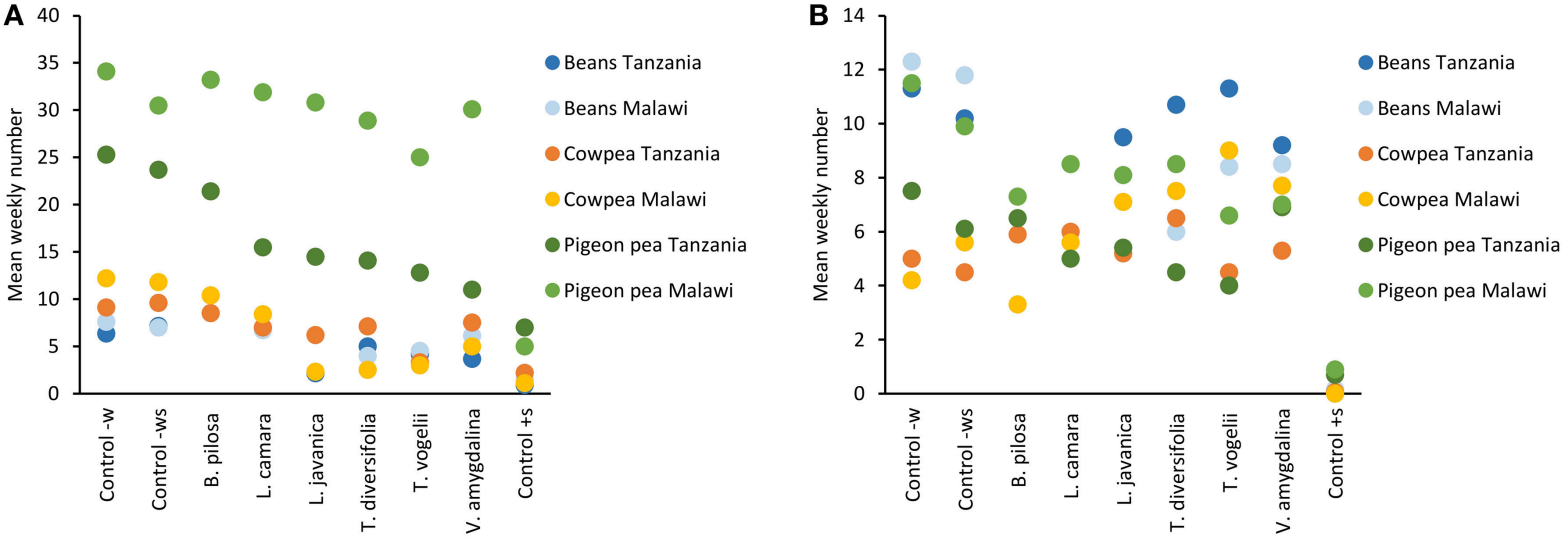
Effect of different pesticidal plant treatments on mean weekly number of **(A)** key indicator pest species (aphids, flower beetles and foliage beetles) and **(B)** key indicator beneficial species (lady beetles, spiders and hoverflies). All plant treatments were applied at 10% w/v with 0.1% liquid soap added to the water during the 24 h extraction period. Control–w is the application of water only, Control–ws is the application of water containing 0.1% liquid soap only, and Control+s is the application of the synthetic pesticide Karate 5 EC (lambda-cyhalothrin pyrethroid, Syngenta) which was applied as per the manufacturers' instructions (20 g/ha).

Field trials carried out across the two countries with three different legume crops over three seasons showed similar trends with respect to pest and beneficial arthropod abundance (Figure 1). The general trend shared across trials was that the positive control synthetic pesticide treatment had very low numbers of both pest and beneficial species, with the negative controls (water only and water with soap) usually having the highest numbers. The pesticidal plant treatments did reduce numbers of both pest and beneficial species but these data more generally followed the abundance observed in the negative controls as opposed to the positive control. An analysis of variance confirms these trends for each crop and location (Table 1). *Lippia javanica, T. vogelii*, and *T. diversifolia* were the most able to reduce pest insect numbers, showing similar effects to the synthetic pesticide in cowpea and bean crops but not for pigeon pea. *Bidens pilosa, L. camara*, and *V. amygdalina* showed similar pest abundance as to that observed in the negative controls. Pest abundance was higher on pigeon pea than on cowpea or bean crops, and this is likely related to the larger size of each plant where pigeon pea grows to ~1.5 m high whereas cowpea and bean crops are <0.5 m high. However, this trend in relative abundance among crops was not strongly observed for beneficial species. Abundance of beneficial species was highest for bean crops, but this was not found to differ significantly from abundance observed in cowpea or pigeon pea crops (Table 1). Beneficial insect numbers in cowpea crops treated with pesticidal plants were higher than that observed in the negative controls, while lower numbers of beneficials were found in bean and pigeon pea when compared to the negative controls. In all crops, the synthetic positive control significantly reduced beneficial numbers compared to all other treatments.

**Table 1:** Treatment effect on abundance of key pest and beneficial arthropod species and legume crop yield during field cropping trials in the countries of Tanzania and Malawi.

**Treatment**	**Beans**						**Cowpea**						**Pigeon pea**					
	**Tanzania**			**Malawi**			**Tanzania**			**Malawi**			**Tanzania**			**Malawi**		
	**Pest abundance**	**Beneficial abundance**	**Yield (kg/ha)**	**Pest abundance**	**Beneficial abundance**	**Yield (kg/ha)**	**Pest abundance**	**Beneficial abundance**	**Yield (kg/ha)**	**Pest abundance**	**Beneficial abundance**	**Yield (kg/ha)**	**Pest abundance**	**Beneficial abundance**	**Yield (kg/ha)**	**Pest abundance**	**Beneficial abundance**	**Yield (kg/ha)**
Control–w	6.3^b^	11.3^b^	1,194.9^c^	7.6^b^	12.3^c^	1,177.1^c^	9.1^c^	5.0^b^	153.2^e^	12.2^c^	4.2^b^	420.8^c^	25.3^c^	7.5^b^	1,446.9^d^	34.1^b^	11.5^b^	439.2^d, e^
Control–ws	7.2^b^	10.2^b^	1,443.2^b, c^	7.0^b^	11.8^c^	1,144.1^c^	9.6^c^	4.5^b^	208.1^e^	11.8^c^	5.6^b, c^	440.9^c^	23.7^c^	6.1^b^	1,726.4^d^	30.5^b^	9.9^b^	613.8^c^
*B. pilosa*	–	–	–	–	–	–	8.5^c^	5.9^b^	501.8^c, d^	10.4^c^	3.3^b^	818.0^b, c^	21.4^c^	6.5^b^	2,351.5^c, d^	33.2^b^	7.3^b^	425.8^d, e^
*L. camara*	–	–	–	6.7^b^	8.5^b^	1,693.1^b^	7.0^c^	6.0^b^	683.9^b^	8.4^c^	5.6^b, c^	729.8^b, c^	15.5^b^	5.0^b^	3,742.4^a, b^	31.9^b^	8.5^b^	375.2^d, e^
*L. javanica*	2.2^a^	9.5^b^	1,753.9^a, b^	–	–	–	6.2^c^	5.2^b^	491.1^d^	2.3^b^	7.1^c^	873.0^b^	14.5^b^	5.4^b^	3,336.1^b, c^	30.8^b^	8.1^b^	644.4^b, c^
*T. diversifolia*	5.0^a, b^	10.7^b^	2,034.7^a^	4.0^b^	6.0^b^	1,830.2^b^	7.1^c^	6.5^b^	709.7^b^	2.5^b^	7.5^c^	876.4^b^	14.1^b^	4.5^b^	3,263.7^b, c^	28.9^b^	8.5^b^	672.3^b, c^
*T. vogelii*	4.2^a, b^	11.3^b^	2,044.9^a^	4.5^b^	8.4^b^	2,337.5^a^	3.3^b^	4.5^b^	1,016.2^a^	3.0^b^	9.0^c^	872.3^b^	12.8^a, b^	4.0^b^	4,464.3^a^	25.0^b^	6.6^b^	812.5^b^
*V. amygdalina*	3.7^a^	9.2^b^	1,575.9^b^	6.1^b^	8.5^b^	2,073.4^a, b^	7.5^c^	5.3^b^	647.1^b, c^	5.0^b, c^	7.7^c^	554.8^b, c^	11.0^a, b^	6.9^b^	3,496.5^a, b^	30.1^b^	7.0^b^	479.5^d, e^
Control+s	0.9^a^	0.1^a^	1,659.6^b^	1.5^a^	0.2^a^	2,327.0^a^	2.2^a^	0.1^a^	1,125.2^a^	1.1^a^	0.0^a^	1,322.5^a^	7.0^a^	0.7^a^	4,407.7^a^	5.0a	0.9^a^	2,285.9^a^
*F*-value	6.1	5.9	14.9	11.7	8.2	34.1	9.5	10.1	111.8	14.3	5.8	10.6	15.6	11.4	24.6	23.2	7.5	28.3
*P*-value	0.0001	0.0001	0.0001	0.0001	0.0001	0.0001	0.0001	0.0001	0.0001	0.0001	0.0001	0.0001	0.0001	0.0001	0.0001	0.0001	0.0001	0.0001

*Abundance values are the average weekly number of three key indicator arthropod species recorded each week over the cropping period. Key pests are aphids, foliage beetles and flower beetles whilst key beneficials are spiders, hoverflies and lady beetles. Values in the same column followed by the same letter are not significantly different from each other at the 95% confidence interval using Tukey's post-hoc Honestly Significant Difference (HSD) test. Control-w, application of water only; Control-ws, application of water containing 0.1% soap, Control+s, application of synthetic pesticide treatment Karate 5 EC (lambda-cyhalothrin)*.

### Crop yield

**Figure 2:**
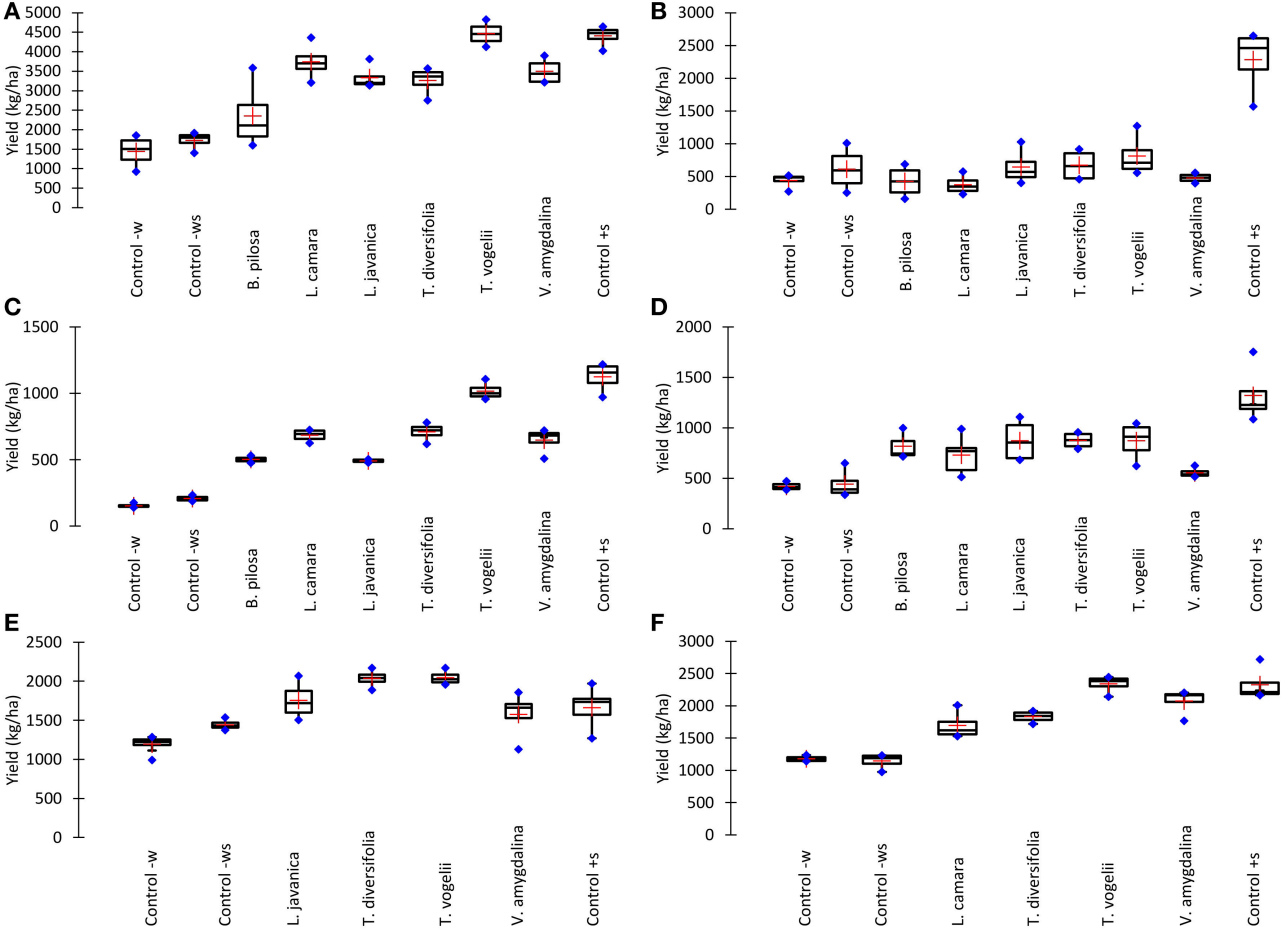
Effect of different pesticidal plant treatments on crop yield of **(A)** Pigeon pea grown in Tanzania, **(B)** Pigeon pea grown in Malawi, **(C)** Cowpea grown in Tanzania, **(D)** Cowpea grown in Malawi, **(E)** Common beans grown in Tanzania and **(F)** Common beans grown in Malawi. All plant treatments were applied at 10% w/v with 0.1% liquid soap added to the water during the 24 h extraction period. Control–w is the application of water only, Control–ws is the application of water containing 0.1% liquid soap only, and Control+s is the application of the synthetic pesticide Karate 5 EC (lambda-cyhalothrin pyrethroid, Syngenta) which was applied as per the manufacturers' instructions (20 g/ha). Boxes represent mean and 95% confidence intervals, blue markers are max. and min. values, orange markers are median values.

Despite insect pest abundance on crops treated with pesticidal plants being significantly higher than observed with the synthetic control, crop yields obtained from pesticidal plant treatments were often comparable to the synthetic pesticide treatment (Figure 2). This is most notable with the use of *T. vogelii* in Tanzania where the yields were statistically comparable for cowpea (1,016–1,125 kg/ha) and pigeon pea (4,407–4,464 kg/ha) and where the bean yield was statistically higher for *T. vogelii* compared to the positive control (2,044 vs. 1,659 kg/ha). *Tephrosia vogelii* treated plants also performed as well as the synthetic pesticide treated on beans in Malawi, but its application on cowpea and pigeon pea produced lower yields than the synthetic in Malawi. Other plant treatments that generally performed well across locations and crops were *L. javanica* and *T. diversifolia*. With the exception of pigeon pea crop yields in Malawi, the negative control treatments (water only, water + soap) had the lowest yields of all the treatments. In some cases, yields of certain pesticidal plant treatments were no better than the untreated controls, notably *B. pilosa* on pigeon pea in Tanzania and *V. amygdalina* on cowpea in Malawi. However, these species did work effectively in other contexts, e.g., *B. pilosa* was effective on cowpea and *V. amygdalina* was effective on bean crops. None of the pesticidal plants appeared to be effective on pigeon pea in Malawi. We suspect this was probably caused by frequent high rainfall in Central Malawi during the 2017 cropping season that inadvertently washed off the pesticidal plant treatments before they were able to affect pests. The synthetic pesticide was still effective on pigeon pea in Malawi, arguably because it was more resistant to frequent rainfall events. An analysis of variance performed on crop yields confirms these observations on yield differences among treatments (Table 1). Comparing the crop yields obtained with the water only application with the different treatments showed that the plant treatments were able to significantly increase the percentage crop yield (Table 2). The simple addition of 0.1% soap to the water generally showed a 20–30% increase in crop yields, most likely due to the well-known effects of soaps on arthropod cuticles and water regulation (Butler et al., 1993). The synthetic control showed the largest percentage increase in yield. However, *T. vogelii* showed comparable yield increases for cowpea and pigeon pea in Tanzania and even exceeded the synthetic on beans in Tanzania. Crop yield will also be affected by the different varieties planted and the slightly different crop spacing employed in each country. Yields are well-known to differ by crop variety (Wallace and Munger, 1966; Talbot, 1984) and other location specific issues such as soil type (Chmelíková et al., 2015), thus preventing statistical analyses of our field trials across locations due to uncontrolled environmental parameters.

**Table 2:** Percentage yield increase when comparing the yield obtained from the untreated control applying water only to the yields obtained from applying the other treatments.

**Treatment**	**Bean yield increase (%)**		**Cowpea yield increase (%)**		**Pigeon pea yield increase (%)**	
	**Tanzania**	**Malawi**	**Tanzania**	**Malawi**	**Tanzania**	**Malawi**
Control–w						
Control–ws	20.8	−2.8	35.8	4.8	19.3	39.8
*B. pilosa*	–	–	227.5	94.4	62.5	−30.6
*L. camara*	–	43.8	346.4	73.4	158.6	−11.9
*L. javanica*	46.8	–	220.6	107.5	130.6	71.7
*T. diversifolia*	70.3	55.5	363.3	108.3	125.6	4.3
*T. vogelii*	71.1	98.6	563.3	107.3	208.5	20.9
*V. amygdalina*	31.9	76.1	322.4	31.8	141.7	−41.0
Control+s	38.9	97.7	634.5	214.3	204.6	376.7

*Control-w, application of water only; Control-ws, application of water containing 0.1% soap; Control+s, application of synthetic pesticide treatment Karate 5 EC (lambda-cyhalothrin)*.

## Discussion

Previous research on the pesticidal plant species used for pest control in this study all have reported bioactivities against insects, parasites, bacteria and fungi (Ganjian et al., 1983; Jisaka et al., 1992; Lina et al., 1992; Pereira et al., 1997; Gu et al., 2002; Rabe et al., 2002; Ogendo et al., 2003; Adedire and Akinneye, 2004; Boeke et al., 2004; Omolo et al., 2004; Kawuki et al., 2005; Koona and Dorn, 2005; Viljoen et al., 2005; Koona et al., 2007; Ambrósio et al., 2008; Asawalam et al., 2008; Mujovo et al., 2008; Oyewole et al., 2008; Bagnarello et al., 2009; Chukwujekwu et al., 2009; Deng, 2009; Gadzirayi et al., 2009; Koul and Walia, 2009; Madzimure et al., 2011; Tesch et al., 2011; Chagas-Paula et al., 2012; Bartolome et al., 2013; Ellse and Wall, 2013; Nhamo et al., 2013; Utono et al., 2014; Green et al., 2017; Kamanula et al., 2017); however, none of these works have investigated the effects of their application on field crop performance or tritrophic impact. Much is also known about the phytochemistry of the six species evaluated. Previous analysis of *L. javanica* has shown the major bioactive component to be camphor, along with minor components including camphene, α-pinene, eucalyptol, Z and E α-terpineol, linalool, cymene, thymol, 2-carene, caryophyllene, and α-cubebene (Mkenda et al., 2015). Camphor has well-documented insecticidal properties (Singh et al., 2014). Chemical analysis of *T. vogelii* has indicated the presence of the rotenoids deguelin, tephrosin and rotenone (with deguelin being the most abundant) (Belmain et al., 2012; Stevenson et al., 2012). Rotenoids are well-known for their anti-insect properties (Ott, 2006). The major compounds in *T. diversifolia* confirmed to have anti-insect properties were identified as the sesquiterpene lactones tagitinin A and tagitinin C (Green et al., 2017). Both compounds were reported recently to be the major compounds in this species (Miranda et al., 2015) while other research indicated tagitinins to have insecticidal activity (Ambrósio et al., 2008). The main anti-insect components of *V. amygdalina* were identified as vernodalin and 11,13-dihydrovernodalin as well as several vernoniosides (Green et al., 2017). Like tagitinin A and C, the first two compounds are sesquiterpene lactones which have exhibited antimalarial, antibacterial and cytotoxic activities (Rabe et al., 2002; Chukwujekwu et al., 2009). The major bioactive components of *L. camara* are germacrene D, β-caryophyllene, a-phellandrene, limonene, and 1,8-cineole (Tesch et al., 2011). Bioactive constituents from *B. pilosa* include β-caryophyllene and τ-cadinene (Deba et al., 2008). *B. pilosa* bioactivity is mainly reported as pharmacological, allopathic, anti-fungal, and anti-bacterial, with no clear anti-insect properties previously noted (Bartolome et al., 2013).

Synthetic pesticides are often misused leading to negative effects on ecosystems and human health, particularly in developing countries (Ecobichon, 2001). Using biocontrol options such as extracts of pesticidal plants has long been argued to be more sustainable and appropriate for smallholder farmers in developing countries (Isman, 2006, 2008; Sola et al., 2014), and our data support this and show that use of pesticidal plants can effectively control pests and be integrated into sustainable agricultural practice. Our data also showed that plant pesticide pest control on legume crops could support yields similar to those on which synthetic pesticides were used. This required relatively frequent weekly application of 10% w/v plant extracts highlighting a trade-off of using pesticidal plants since the active components break down quickly and have low persistence (Casida, 1980). This means that labor inputs must increase when using crude preparations of pesticidal plants, although the commercialization of botanical products that incorporate photostabilizers and sticking agents could prolong their efficacy on crops. This sort of trade-off is generally accepted by many smallholder farmers because using synthetic pesticides requires financial outlay, whereas using pesticidal plant materials simply requires labor costs for harvesting and processing. Economic analyses of pesticidal plant use in Africa generally show that they are more cost-beneficial for smallholder farmers than using synthetic pesticides as pesticidal plant use reduces input costs, even when costing in extra labor, with generally little yield loss trade-off (Amoabeng et al., 2014; Mkenda et al., 2015). On the other hand lower persistence of pesticidal plants means that the health of consumers is at less risk owing to reduced exposure to bioactive compounds from the plants which decompose into harmless natural products unlike the synthetic compounds that persist in and on plants for weeks or in soil for months or years. This means that crops can be harvested without the risk of residues remaining due to the rapid breakdown of naturally occurring compounds when exposed to UV light, and micro-organisms in soil/water (Isman, 2000; Angioni et al., 2005; Caboni et al., 2006). Another benefit for smallholder farmers is that it enables production for higher value organic markets and for export.

The research presented highlights another important trade-off when comparing synthetic vs. pesticidal plant crop protection effects on non-target species. The persistence and generic toxicity of synthetic pesticides inevitably means their impact on pollinators, predators, and parasitoids is usually very high (Potts et al., 2010; Stanley and Preetha, 2016). Indeed, our research showed that a synthetic pesticide commonly used on legume crops and other horticultural produce resulted in the absence of nearly all beneficial indicator species monitored over the cropping season. This typically leads to the phenomenon of pest resurgence once the synthetic pesticide wears off enabling pest species populations to expand in the absence of predatory species (Roubos et al., 2014; Welch and Harwood, 2014). However, the pesticidal plant treatments had much less of an impact on indicator beneficial species. Some of the plant materials did reduce the numbers of beneficials in comparison to the untreated (water and water+soap) controls, but in all cases these reductions were not as severe as that observed in the synthetic treatment. Overall, there was very little difference among the pesticidal plant treatments and the untreated controls in terms of beneficial numbers. We expect this is partly due to lower persistence of plant treatments but also due to different modes of action where the plant treatments may be acting against pests as repellents, anti-feedants or through toxicity post-ingestion. Lower toxicity and persistence of the pesticidal plant treatments is supported through their reduced effects on indicator pest species. In only a few cases were the pesticidal plant treatments just as successful as the synthetic in reducing pest abundance. Pest and beneficial species were less affected by the pesticidal plant treatments compared to the synthetic. We would argue that this would help natural pest regulation manage pest numbers more effectively because natural pest regulation is most effective when the ratio between pest and predator numbers is low (Arditi and Ginzburg, 1989; Rusch et al., 2010). The continual knock-down provided by the pesticidal plant treatments allows the beneficial species to contribute to pest regulation at a meaningful scale (Crowder et al., 2010). The protection and facilitation of ecosystem services provided by pollinators, predators and parasitoids through using pesticidal plants is a strong argument for the adoption of pesticidal plant extracts in crop protection.

Despite higher numbers of pests on the pesticidal plant treatments compared to the synthetic treatment, yields were often comparable. This could be due to further pest reduction through natural enemies. However, this may also be because plant species can tolerate a certain amount of damage and are able to physiologically compensate to maintain overall yield (Rubia et al., 1996; Brown, 2005). Compensation usually requires that plants are generally in good health, with access to sufficient nutrients and water, with little other sources of stress (Tardieu and Tuberosa, 2010). Although we did not measure such responses, legume crop compensation may have been facilitated through the frequent application of pesticidal plant extracts. This could involve other forms of plant protection by direct control of bacterial or fungal pathogens (Soylu et al., 2010; Marei et al., 2012; Rasoul et al., 2012), or indirect physiological assistance by acting as a topical green fertilizer (Jama et al., 2000), bio-stimulant (Pretali et al., 2016), or foliar feed (Shaaban, 2001). We are undertaking further field trials to assess the multiple benefits of using pesticidal plants for smallholder crop production, which should provide more evidence for their integration in to agro-ecologically sustainable crop production systems.

## Author contributions

SB, PS, YT, and PN conceived the study. AM, PM, RM, and NM were involved in the study design. SB carried out the statistical analysis and wrote the first draft of the manuscript. YT, AM, PM, RM, and NM carried out field trials and data collection. All authors were involved in writing the manuscript, and gave final approval for publication.

### Conflict of interest statement

The authors declare that the research was conducted in the absence of any commercial or financial relationships that could be construed as a potential conflict of interest.
